# Predicting microbiomes through a deep latent space

**DOI:** 10.1093/bioinformatics/btaa971

**Published:** 2020-12-07

**Authors:** Beatriz García-Jiménez, Jorge Muñoz, Sara Cabello, Joaquín Medina, Mark D Wilkinson

**Affiliations:** Centro de Biotecnología y Genómica de Plantas (CBGP, UPM-INIA), Universidad Politécnica de Madrid (UPM) - Instituto Nacional de Investigación y Tecnología Agraria y Alimentaria (INIA), Campus de Montegancedo-UPM, 28223-Pozuelo de Alarcón, Madrid, Spain; Serendeepia Research, 28905 Getafe (Madrid), Spain; Centro de Biotecnología y Genómica de Plantas (CBGP, UPM-INIA), Universidad Politécnica de Madrid (UPM) - Instituto Nacional de Investigación y Tecnología Agraria y Alimentaria (INIA), Campus de Montegancedo-UPM, 28223-Pozuelo de Alarcón, Madrid, Spain; Centro de Biotecnología y Genómica de Plantas (CBGP, UPM-INIA), Universidad Politécnica de Madrid (UPM) - Instituto Nacional de Investigación y Tecnología Agraria y Alimentaria (INIA), Campus de Montegancedo-UPM, 28223-Pozuelo de Alarcón, Madrid, Spain; Centro de Biotecnología y Genómica de Plantas (CBGP, UPM-INIA), Universidad Politécnica de Madrid (UPM) - Instituto Nacional de Investigación y Tecnología Agraria y Alimentaria (INIA), Campus de Montegancedo-UPM, 28223-Pozuelo de Alarcón, Madrid, Spain; Departamento de Biotecnología-Biología Vegetal, Escuela Técnica Superior de Ingeniería Agronómica, Alimentaria y de Biosistemas, Universidad Politécnica de Madrid (UPM), Madrid, Spain

## Abstract

**Motivation:**

Microbial communities influence their environment by modifying the availability of compounds, such as nutrients or chemical elicitors. Knowing the microbial composition of a site is therefore relevant to improve productivity or health. However, sequencing facilities are not always available, or may be prohibitively expensive in some cases. Thus, it would be desirable to computationally predict the microbial composition from more accessible, easily-measured features.

**Results:**

Integrating deep learning techniques with microbiome data, we propose an artificial neural network architecture based on heterogeneous autoencoders to condense the long vector of microbial abundance values into a deep latent space representation. Then, we design a model to predict the deep latent space and, consequently, to predict the complete microbial composition using environmental features as input. The performance of our system is examined using the rhizosphere microbiome of Maize. We reconstruct the microbial composition (717 taxa) from the deep latent space (10 values) with high fidelity (>0.9 Pearson correlation). We then successfully predict microbial composition from environmental variables, such as plant age, temperature or precipitation (0.73 Pearson correlation, 0.42 Bray–Curtis). We extend this to predict microbiome composition under hypothetical scenarios, such as future climate change conditions. Finally, via transfer learning, we predict microbial composition in a distinct scenario with only 100 sequences, and distinct environmental features. We propose that our deep latent space may assist microbiome-engineering strategies when technical or financial resources are limited, through predicting current or future microbiome compositions.

**Availability and implementation:**

Software, results and data are available at https://github.com/jorgemf/DeepLatentMicrobiome

**Supplementary information:**

Supplementary data are available at *Bioinformatics* online.

## 1 Introduction

### 1.1 The microbiome

Microbes are everywhere, in human, animals, plants, etc., executing numerous biological functions whose absence would dramatically reduce the quality and quantity of life on earth (Gilbert and Neufeld, 2014). Study of microbial communities has increased in recent years due to advances in high-throughput technologies that now allow identification of microbes in a community by sequencing rather than culturing (Lloyd-Price *et al.*, 2017; Thompson *et al.*, 2017). Microbial community functions include collaborating in carbon and nitrogen cycles, to provide nutrients by breaking complex molecules into smaller compounds, training and triggering the immune system to fight against pathogens, etc. Those microbiome functions entail applications in health and medicine, climate change, sustainable agriculture, environment and biofuels.

Most microbiome analyses to date have focused on observational or descriptive approaches; i.e. to identify the microbes living in a community and to establish correlations between those experimental findings and some phenotypic feature, such as abundance or deficiency of a particular strain in a disease’s subject group. For example, comparing human gut in health versus gastrointestinal disease subjects (Kotloff *et al.*, 2013) or the soil microbiome before and after a large environmental challenge (Uritskiy *et al.*, 2019). Some microbiome studies go one step further, following a predictive approach (Zhou and Gallins, 2019). These translational approaches should allow us to design solutions based on microbiome modulation to address problems in human and plant health, and take microbial composition as a predictor of a particular phenotypic feature.

### 1.2 ML in microbiome research

Studies focused on the microbiome and metagenomics, where Machine Learning (ML) approaches are applied, have recently grown in number (Sakowski *et al.*, 2019). We distinguish two major types of study. First, single problem studies, e.g. microbial composition being used to predict productivity in soil (Chang *et al.*, 2017), contaminants and geochemical features in wells (Smith *et al.*, 2015), presence/absence of disease due to changes in abundances of microbes over time (Bogart *et al.*, 2019) or biomarkers of cancer from the human blood microbiome (Poore *et al.*, 2020). Second, global methods, where the same tool may be applied to multiple, distinct datasets with alternative predictive goals. For example, MetAML (Pasolli *et al.*, 2016), a tool for metagenomics-based prediction tasks and for microbiome-phenotype associations; q2-sample-classifier (Bokulich *et al.*, 2018), a plugin for QIIME 2 for supervised classification; Microbiome Learning Repo (Vangay *et al.*, 2019), which provides a benchmark of 33 curated classification and regression tasks from 15 published human microbiome datasets, and SIAMCAT (Wirbel *et al.*, 2020), some versatile ML workflows in a R package.

### 1.3 Deep learning in bioinformatics and microbiome/metagenomics

Recently, there has been an emergence of Deep Learning (DL) approaches (Lecun *et al.*, 2015) to biological challenges (Ching *et al.*, 2018; Min *et al.*, 2017). When the input data are images or sequences (i.e. DL propitious data formats), DL improved performance in areas, such as medical image-based diagnostics, and predictions based on genomic sequence analysis (Li *et al.*, 2019). DL also contributes to improvements in data representation or automatic feature extraction, e.g. retrieving features from Electronic Health Records to calculate a patient’s risk of disease (Miotto *et al.*, 2016). Heterogeneous autoencoders have been previously proposed, e.g. to integrate sequential and non-sequential data, to manage the sparsity problems from recommender systems (Li *et al.*, 2018) and to provide novel co-training algorithms for architectures (Xu *et al.*, 2020).

With respect to microbiome studies, DL has not yet been as widely applied compared to other bioinformatics problems. Examples are limited: phenotype prediction from bacterial composition with long short-term memory (food allergy) (Metwally *et al.*, 2019) or convolutional neural network (disease) (Sharma *et al.*, 2020), human age prediction (Galkin *et al.*, 2020), identification of body-site and prediction of Crohn’s disease with a *k*-mer representation (Asgari *et al.*, 2018), identification of microbiome biomarkers using graph embedding (Zhu *et al.*, 2019) or prediction of metabolites with autoencoders (AEs) (Le *et al.*, 2020). Generally speaking, phenotype prediction from metagenomics data is the most common task solved by M/DL in the microbiome space (LaPierre *et al.*, 2019).

### 1.4 Our proposal

Pursuing the promising translational and predictive approach, in this work, we propose an ambitious goal beyond predicting phenotypic features from microbial composition. Rather, we attempt to achieve the opposite: to predict the microbial composition based on a few phenotypic and/or environmental features. Our approach would be useful, e.g. in cases where one must make a decision whose outcome depends on microbial composition, but where sequencing facilities are not available, sequencing costs are prohibitive, or where the only data available are phenotypic/environmental features. For example, a farmer in an emerging economy, with no access to sequencing facilities (nor finances to engage them), might want to make strategic decisions about what crop to cultivate on their land, or which fertilizer to use in what quantity—a decision often highly dependent on that soil’s microbial composition.

Few prior studies have attempted to predict microbial composition from environmental features, and those have focused primarily on a few tens of taxa, and at the highest taxonomic levels (Ladau *et al.*, 2018; Larsen *et al.*, 2012; Oh and Zhang, 2020). We provide a detailed comparison with these similar approaches in the Supplementary Material.

### 1.5 Our approach and contributions

The primary contributions of this work are: (a) the development of a novel AE model that merges knowledge from both microbial composition and environmental/mapping features, into a deep latent space; (b) an exhaustive selection and evaluation of AE reconstructions of the whole microbial composition (hundreds of taxa) from that latent space; and (c) prediction of microbiome composition starting from only environmental features. All of these contributions are supported by real data from a soil microbiome study, in particular, the Maize rhizosphere microbiome. Maize is an important food crop, in a world reaching an estimated 10 billion people by 2050. This will require a doubling of food production on scarce agriculture soil, with increasingly limited water, and avoiding the expensive and destructive application of fertilizers (Hunter *et al.*, 2017). As such, agrifood research—the focus of this work—is of great interest and socioeconomic importance.

While the initial application of our system is aimed at predicting the microbiome based on a limited number of environmental features, we believe that the system could also be applied to the prediction of the microbiome for novel or hypothetical ecosystems. This would help us prepare for the consequences of environmental influences, such as climate change, or toxic spills. In addition, our system can be applied to predict the microbiome of novel datasets, with a limited number of samples or observations, via transfer learning.

## 2 Materials and methods

### 2.1 Dataset

We selected the soil microbiome of an agronomically important plant (*Zea mays* L. subsp. mays), using a dataset taken from the Walters *et al.* (2018) study. The authors examined the influence of the soil microbiome on the traits of maize to clarify whether the surrounding microbial communities could be used as a breeding trait. This 16S rRNA dataset includes multiple cropping fields, and includes 27 different maize varieties.

Our dataset consists of 4724 samples × 717 operational taxonomic units (OTUs) × 5 environmental features. Samples were split into training: testing (90%:10%) sets, and within the training set there was 5-fold cross-validation (CV).

The environmental features were: temperature, precipitation (accumulation 3 previous days), plant age, maize line and maize variety. We selected these variables based on their likelihood of being applicable to novel datasets through excluding constant, redundant, sample-dependant and geographical location features, thus ensuring the predictor is sufficiently general. Metadata about temperature and precipitation was kindly provided to us by the authors as it was not included in their online repository (qiita ID: 11116).

We also utilized a smaller dataset from the Maarastawi *et al.* (2018) study, focused on Italian and Philippine maize rhizosphere microbiomes. This small dataset was selected to become our transfer learning proof-of-concept. The original Maarastawi dataset has 322 samples and 1943 bacterial OTUs. After filtering out samples from bulk soil, and filtering for missing values in metadata, 123 samples remained. Regarding OTUs, due to lack of standardization in microbiome taxa annotation, it was infeasible to map those 1943 OTUs to the 717 OTUs identified in the Walters *et al.* (2018) dataset. There was limited overlap at low taxonomic levels (26/45 Class, 36/83 Order, 70/144 Family, 84/222 Genus and 0/717 Species level). Our final dataset therefore included only 15 phylum-level OTUs, shared between the two studies, to be the target of transfer learning studies. The mapping features correspond primarily to chemical soil properties (pH, Nitrogen and Carbon concentrations, clay fraction, soil type and water holding capacity). Samples were split into training: testing (70%:30%) sets.

### 2.2 Model architectures

**Fig. 1.**
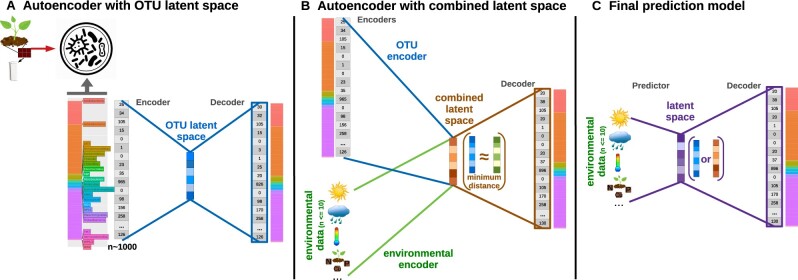
Schema of AE and final model architectures. (**A**) AE architecture with an OTU latent space. (**B**) AE architecture with a combined latent space (brown), which minimizes the distance between OTU (blue) and environmental (green) latent spaces during model training. (**C**) Final prediction model with environmental features as input, where the latent space and the decoder could come from AE in panel A (OTU latent space) or AE in panel B (combined one)

Our DL models follow an AE architecture. As such, we first design an encoder model that transforms the input features (in this case, microbial abundances and/or environmental features) to a latent space. Subsequently, we design a decoder model that predicts the output (in this case, the microbial composition) from the latent space (Fig. 1). The latent space is an encoded representation of the input features that (generally) reduces their dimensionality; e.g. in our case, from >700 OTUs plus the environmental variables down to 10 values. Subsequently, the problem of predicting microbial composition at low taxonomic levels, using only a few environmental features, becomes attainable. Those latent space values are non-linear combinations of the input features.

We defined two encoders: (i) OTU composition → latent space, (ii) environmental variables → latent space; and one decoder model: latent space → OTU composition. With those modules, we then designed two different AE architectures. In one AE, the latent space depends only on the OTU composition (Fig. 1A). The second AE includes both encoders, which share the same decoder. During training, the latent space of both encoders is forced to be similar—i.e. the difference between the two latent spaces is minimized as part of the loss function, to create a latent space for the environmental variables as similar as possible to the OTU latent space, and vice versa (Fig. 1B). The final module of the architecture is a predictor that returns a latent space (OTU or combined) from environmental features. From there, the final prediction model integrated a predictor and that decoder (Fig. 1C). So, both latent spaces allow prediction of the OTU composition using only environmental features—the final goal of this study.

### 2.3 Transfer learning

Transfer learning is a learning framework, which consists of re-using a model trained with sufficient samples (large dataset) in a different but similar scenario where there are too few samples to undertake training (small dataset) (Pan and Yang, 2010).

### 2.4 Parameterization: model training and selection

The hyperparameters of the DL model were selected according to the evaluation metrics over a 5-fold CV within the training dataset.

Regarding normalization, we used Total-Sum normalization (TSS) and TSS followed by Centered Log Ratio (CLR), both approaches are per-sample normalizations and suitable for 16S microbiome absolute abundance data (Weiss *et al.*, 2017) and for managing the compositionality of microbiome data (Zhou and Gallins, 2019). We used distinct loss functions to guide the training: mean squared error (MSE), Crossentropy and Bray–Curtis dissimilarity. Our normalization approaches and loss functions are detailed in the Supplementary Material.

Beyond the normalization and loss functions described above, the other parameters whose values were tuned were: the latent space size (10, 50, 100); number and size of the hidden layers [(512, 256), (256), none]; the activation function of the encoder and decoder (tanh, relu, sigmoid) and of the latent layer (tanh, sigmoid); the learning rate (0.01, 0.001) and the batch size (64, 128). Additional details are provided in the Jupyter notebook documenting this study, available on GitHub to allow for review and reproduction of our results.

### 2.5 Evaluation metrics

We use several kinds of metrics to assess the performance of our prediction model, comparing the predicted versus actual microbial composition. First, the most common metrics used in regression learning tasks to assess reconstruction error in AEs are the mean absolute error (MAE), mean squared error (MSE) and mean absolute percentage error (MAPE). However, these are difficult to interpret (beyond ‘the lowest’ and ‘the best’) and in this case would be difficult to compare because we apply different normalization approaches, confounding these scale-dependent metrics. As such, we compute additional scale-independent metrics, such as Pearson correlation (mean correlation between each predicted and actual OTU across all samples), that are similarly used by other bio-AE systems (Manica *et al.*, 2019). An additional metric selected was the Bray–Curtis dissimilarity, described above.

To sort the best-predicted OTUs, we use the root relative squared error (RRSE)—a scale-independent error metric that could be applied to individual OTUs, rather than samples, and is suitable for comparing variables with large differences in values, as seen in the relative abundances of different taxa.

## 3 Results

### 3.1 Microbial composition is predicted with high accuracy from environmental features

Table 1 shows a quantitative evaluation of our proposed model to satisfy our main goal: to predict the 717 OTU abundances within a maize root microbiome starting from environmental variables.

**Table 1.** Performance of evaluation metrics. In the test set

	Default		Linear regression		MLP		OTU latent space		Combined latent space	
Input Variables	Pearson	Bray–Curtis	Pearson	Bray–Curtis	Pearson	Bray–Curtis	Pearson	Bray–Curtis	Pearson	Bray–Curtis
Age, T, rain, line, variety	0.5852	0.5140	0.6629	0.4659	0.7219	0.4169	**0.7340**	0.4222	0.7229	**0.4065**
Age, T, rain	0.5852	0.5140	0.6641	0.4638	0.6927	0.4527	**0.7348**	0.4181	0.7220	**0.4072**
T and rain	0.5852	0.5140	0.5881	0.5089	0.6323	0.5047	0.6087	0.4686	**0.6676**	**0.4557**
Age and T	0.5852	0.5140	0.6622	0.4664	0.6814	0.4591	**0.7323**	0.4204	0.7189	**0.4094**
Age and rain	0.5852	0.5140	0.6628	0.4728	0.7155	0.4211	**0.7361**	0.4200	0.7048	**0.4139**

*Note*: In Pearson, higher scores are better, because it is a correlation metric. In Bray–Curtis, lower scores are better, as it is a dissimilarity metric. Bold means the best model per metric and row. Underline means the best model per metric in the table.

Given the absence of prior-art or a gold standard for this kind of prediction, we compare the accuracy of our models with three baseline models capable of simultaneous regression of multiple variables: (i) a default predictor averaging microbe abundances (per OTU) over all training samples, returning a consistently identical output regardless of the mapping feature; (ii) a linear regression model; (iii) a non-linear model, i.e. a multi-layer perceptron (MLP). In cases (ii) and (iii), there is neither latent space, nor an AE; they represent alternative approaches to predicting OTU abundances from environmental features.

Table 1 shows that our AE models with latent space (last two grouped-columns) outperform baseline models (first three grouped-columns) in both correlation (e.g. AE models have higher Pearson correlation than non-AE) and community ecology metrics (e.g. the combined model always displays the lowest Bray–Curtis dissimilarity). Notably, OTU latent space models exhibit the best performance.

Among the multiple models tested, on the basis of its performance, we selected the OTU latent space model taking three variables (plant age, rain and temperature). This is our reference model for the remainder of the analyses in this study. It is the model with the second-best Pearson correlation (with only a slight difference of 0.0013 between first and second-best) and the best Bray–Curtis score among all the OTU latent space models. OTU latent space exhibits the best Pearson correlation for almost all subsets of environmental features (i.e. rows), though the best Bray–Curtis is achieved by the combined latent space models.

### 3.2 Hyperparameters yielding the highest prediction accuracy could be identified

We identified the combination of hyperparameters resulting in the best model to predict microbial composition, based on a standard validation with a 5-fold CV on the training set.

Our criteria in selecting the optimal model were to both maximize the Pearson correlation and to minimize Bray–Curtis dissimilarity. The model with the highest Pearson correlation (0.7390 and 0.4204 in Bray–Curtis) corresponded to an architecture with a latent space size of 100. In terms of community ecology, the best model has 0.7384 Pearson and 0.4015 Bray–Curtis, with a latent space of 100. Finally, the best model among those having the smallest latent space size (10) achieves a similar performance to those just described (0.7203 Pearson and 0.4123 Bray–Curtis). As such, in terms of a simpler encoded representation, we selected this as the overall reference model, and used it in the remaining analyses. The detailed parameters and evaluation of the three models described above are in GitHub (experiments 188, 366 and 351 in the ‘autoencoder_results’ Jupyter notebook).

**Fig. 2.**
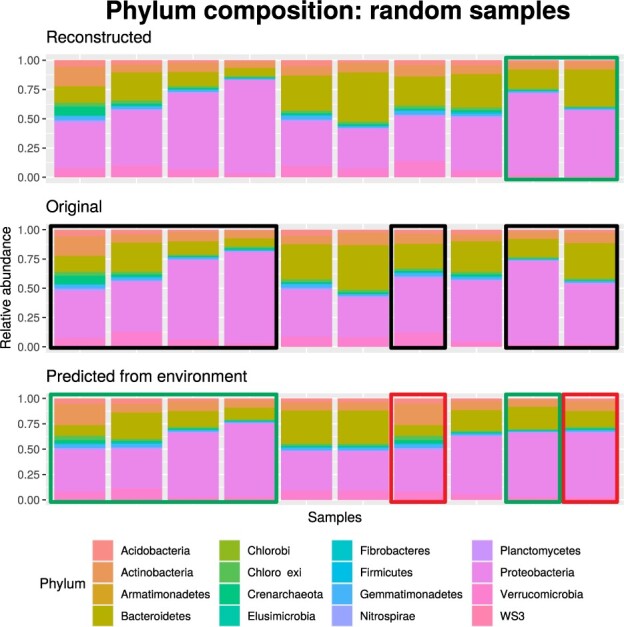
Example of reconstruction and prediction of microbial composition. In the center row is the original microbial composition, allowing it to be compared to both the reconstructed (top) and that predicted from environmental features (bottom). One sample per column. Each Phylum taxonomic category is assigned a different color. Green/red boxes highlight examples of good/bad sample reconstructions or predictions, and their corresponding original microbial composition is denoted with black boxes

We have also analyzed the performance of the reconstruction of the microbial composition from the latent space by our AE. As expected, the performance of the reconstruction alone is better than the performance of the prediction from environmental features (see Fig. 2). For example, reaching 0.9174 in Pearson correlation and 0.2065 in Bray–Curtis dissimilarity in 5CV in the reference selected model. Supplementary Figure S1 shows the learning curves of the AE trained for that reconstruction, both for OTU and combined latent spaces.

Regarding the remaining parameters, with respect to normalization, TSS and CLR perform similarly. The larger the latent space the better, though there is only a 0.01 point difference in Pearson correlation between sizes 50 and 100. We saw the best results with larger AEs (more hidden layers, more nodes per layer). We noted little difference between the distinct activation functions. Finally, a batch size of 64 with a learning rate of 0.001 resulted in higher accuracy than a batch size of 128 with a learning rate of 0.01, probably due to the small dataset size used for DL.

The results of the 405 combinations of parameters, with exhaustive metric computations, are summarized in Supplementary Figure S2, and can be examined in detail in the available ‘autoencoder_results’ notebook on GitHub.

### 3.3 Plant age and rain are the most relevant features to predict microbial composition

The performance of different subsets of variables points out that plant age and rain is the combination of features exhibiting the best performance (0.7361 Pearson Correlation) (Table 1, across rows).

To complement this relevant-features analysis, we trained individual models where the input is only one feature (detailed results available on GitHub). This analysis highlights plant age is the most important feature (0.7330 Pearson), followed by rain (0.6673), temperature (0.6064) and finally inbreds and the maize line (<0.58).

Plant age and rain as relevant variables associated to microbial composition are in agreement with the original publication (Walters *et al.*, 2018), although the authors focus in that study was to find relevant features that enable discovery of heritable OTUs, rather than to predict OTU abundances. The influence of precipitation on the maize microbiome is also noted by Tan *et al.* (2020), indicating a strong correlation between rain and the maize soil bacterial diversity.

### 3.4 *Actinobacteria*, *Acidobacteria* and *Proteobacteria* were the most accurately predicted taxa

This section explores and interprets the predictive model, providing a qualitative evaluation of the best microbial composition prediction.

Figure 2 shows a comparison of the original (center) versus the reconstructed (top) and environmental-feature-predicted microbial composition (bottom) over a demonstrative subset of samples in the test set. See figures for all the samples in the test set, grouped by maize line or variety, on GitHub (‘Results’ folder), and Supplementary Figure S3 summarizing the reconstruction performance for all samples.

In general, Figure 2 shows a trend toward preserving the pattern of microbial composition (in the figure shown as a similar color distribution pattern) among the three vertical blocks—reconstructed, original and predicted. That tendency is more pronounced in the reconstructed versus the original microbial composition (top and central blocks). For example, the last two samples on the right are very accurately reconstructed (double green box), distinguishing the higher relative abundance of *Bacteroidetes* in the last column. Several other samples, grouped on the left of the figure, show more accurate predictions, as indicated by the green boxes. However, there are samples where the predictions are less accurate (red boxes), e.g. the last sample on the right, where the abundance of *Bacteroidetes* is particularly badly-predicted. The other red box in Figure 2 highlights another badly-predicted sample, with a higher relative abundance of *Actinobacteria* (orange) in the predicted compared to the original microbiome, and a loss of abundance of *Bacteroidetes*, *Proteobacteria* (purple) and *Verrucomicrobia* (pink).

In the top 5% predicted OTUs (sorted by ascending RRSE), *Actinobacteria* are the most abundant Phylum group (31.43%), followed by *Acidobacteria* and *Proteobacteria* (22.86% each), *Verrucomicrobia* (8.57%), *Bacteroidetes* and *Planctomycetes* (5.71% each) and *Chloroflexi* (2.86%). Comparatively, the composition of OTUs in the test set maize root microbiome has the most abundant Phyla as *Proteobacteria, Actinobacteria* and *Bacteroidetes* (39.19%, 18.83% and 13.39% respectively). Thus, *Bacteroidetes* is a group of taxa under-represented in the best-predicted OTUs (5.71%) compared to reality (13.39%); and, conversely, *Acidobacteria* is a group of taxa over-represented in the best-predicted OTUs (22.86%) compared to the original OTU data (9.21%).

The 10 best-predicted OTUs are (in terms of their Order-level taxonomy): *iii1-15/RB40, MND1, Rhizobiales, 0319-7L14, Ellin6067, Pirellulales, Gaiellales, Actinomycetales, iii1-15* and *Acidimicrobiales*.

### 3.5 Microbiome prediction improves at higher taxonomic levels

Table 2 shows the performance of the prediction of microbial composition when the taxa are aggregated to a higher taxonomic level, reducing the number of OTUs that must be predicted from hundreds to tens. As expected, the higher the taxonomic level (top of the table), the better the performance (i.e. higher Pearson score and lower Bray–Curtis metric). The highest differences are between Phylum and Class, and between Genus and Species.

**Table 2.** Performance at different taxonomic levels

		OTU latent space		Combined latent space	
Taxonomic level	No. OTUs	Pearson	Bray–Curtis	Pearson	Bray–Curtis
Phylum	16	0.9576	0.1591	0.9451	0.1833
Class	45	0.8777	0.2514	0.8646	0.2610
Order	83	0.8264	0.3007	0.7983	0.3057
Family	144	0.8229	0.3239	0.7965	0.3229
Genus	222	0.8133	0.3414	0.7901	0.3408
Species	717	0.7348	0.4181	0.7220	0.4072

*Note*: Based on reference model configuration, with the three selected input variables.

### 3.6 Predicting the microbiome of novel or hypothetical ecosystems

This section describes how our model is able to predict the microbiome of a hypothetical ecosystem, e.g. where the environmental features have been defined based on a predicted future environmental state.

**Fig. 3.**
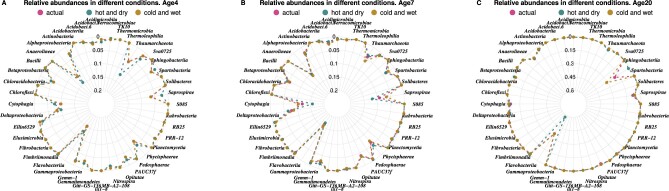
Prediction of microbial composition in different predicted climate change conditions, at distinct plant ages. Outcomes are reported at the Class taxonomic level. Each colored point and dashed line indicates a sample in a different temperature/precipitation condition. ‘actual’: 59°F and 1.5 inches of rain; ‘hot and dry’: 86°F and 0 inches of rain; ‘cold and rain’: 50°F and 5 inches of rain. Note the difference in the maximum of relative abundance between A/B (0.2) and C (0.6)

We designed two challenge scenarios, with the goal of simulating climate change conditions—i.e. modifying temperature and humidity. First, we predicted the microbiome in a soil influenced by conditions of higher temperature and lower precipitation (labeled ‘hot and dry’ in Fig. 3). Second, we predicted the soil microbiome in ‘cutoff low’ conditions (i.e. low pressure centers aloft) characterized by heavy rains and lower temperatures (labeled ‘cold and wet’ in Fig. 3).

Figure 3 compares the relative abundances of the microbial composition in three different conditions: an ‘actual’ situation with intermediate values of temperature and precipitation, and two other scenarios with very high or very low temperature and/or rainfall. Relevant events can be identified when the taxa exhibit divergent relative abundance (i.e. the points in Fig. 3 do not overlap), thus corresponding to taxonomic abundance changes predicted to be due to distinct changes in temperature or humidity.

When we analyzed changes at different plant ages, at an age of 4 weeks (Fig. 3A), in ‘hot and dry’ conditions associated to global warming, our model predicts a microbiome with a higher relative abundance of *Thermoleophilia*, *Alphaproteobacteria* and *Deltaproteobacteria* (green point nearer the center of the radar graph) and a lower relative abundance (i.e. further from the center) of *Saprospirae*, *Cythophagia*, *Gammaproteobacteria* and *Pedosphaerae*. This prediction regarding changes under global warming conditions are consistent with previous experimental data about soil microbiomes in arid zones (Liu *et al.*, 2018), where *Thermoleophilia* and *Alphaproteobacteria* appears among the high abundance taxa, and *Gammaproteobacteria* and *Deltaproteobacteria* as low abundance taxa (the latter is the only observation not in agreement with that study). In ‘cold and wet’ conditions, the changes in relative abundances versus actual conditions are less pronounced than in the ‘hot and dry’ condition (the pink and brown points generally overlap or are close). Figure 3B represents the trend of changes in relative abundances of soil microbiome with maize plants at age 7 weeks, under distinct temperature and humidity conditions. Some taxa show a relative abundance gradient over plant age. For example, the relative abundance of *Cythophagia* and *Saprospirae* in ‘hot and dry’ conditions progressively increase into the second month, and eventually become more abundant than the same taxa in the other conditions. In older plants, at 16 or 20 weeks of age (Fig. 3C), the relative abundance gradient increases in *Gammaproteobacteria* in ‘hot and dry’ conditions (green), and in *Sphingobacteria* in the ‘cold and wet’ (brown). Additional examples can be seen in Supplementary Figure S4.

### 3.7 Transfer learning

In this section, we predict, starting with very few samples, the microbial composition of a maize-associated soil. Our objective is to evaluate the utility of our latent space approach, via transfer learning, in scenarios with a limited number of sequenced microbiome samples (<100 being a typical case) and/or a scarce number of measured variables, where *de novo* construction of a predictor would be difficult due to lack of data. In such a scenario, transfer learning from an existing large dataset, onto a smaller, sparser dataset, would have great utility.

The knowledge transferred is stored in the latent space and its decoder. The transfer learning model has, as its first module, a new predictor that takes the environmental features as input and generates a latent space prediction as output. The second module in the transfer learning model is the decoder, generating the microbial composition from the latent space; i.e. the decoder that was built with the Walters *et al.* training dataset, where the transfer learning knowledge resides.

Our first test of transfer learning was to use the knowledge stored in our deep latent space, built using a large sample-size, to predict the maize root microbiome from a hypothetical small farm with limited sequencing resources. The hypothetical objective would be that they could be guided toward taking appropriate, contextually-sensitive action (e.g. fertilization, addition of biotics, etc.). To simulate this scenario, we took a subset of 100 samples from the Walters *et al.* (2018) dataset (not used previously, neither in training nor test). With that small dataset, we then build a latent space predictor from the environmental features as input and, in the second module, the decoder takes the latent prediction as input to generate a microbial composition prediction. In this case, the input variables of the latent predictor are the same as those in the model used for the knowledge transfer: temperature, rain and plant age; and the output being the 717 OTUs.

A second test of transfer learning is to assume that the smaller dataset has entirely different environmental features compared to the features in the primary model. To model this scenario, we used the Maarastawi *et al.* (2018) maize root microbiome dataset. That study did not collect environmental features about weather, but rather about the chemical composition of the soil; as such, the Maarastawi prediction models use nitrogen and carbon concentration, together with pH. In this case, a new latent space predictor is built that takes these three new variables as input. The rest of the transfer learning model remains the same (i.e. applying the previously-built decoder in the second module).

In both transfer learning scenarios, our AE models resulted in better performance than the baseline predictors (Table 3). In particular, the model using only the OTU latent space performed best.

**Table 3.** Prediction performance with transfer learning from the primary model to smaller datasets

	Walters *et al.*’s subset		Maarastawi *et al.*	
	Pearson	Bray–Curtis	Pearson	Bray–Curtis
Linear regression	0.5436	0.5596	0.1588	0.6437
MLP	0.7114	0.4410	0.7230	0.3347
OTU latent sapce	**0.7544**	**0.4280**	**0.7654**	**0.3233**
Combined latent space	0.7266	0.4346	0.7060	0.3728

*Note*: Walters *et al.* (2018)’s subset: 100 samples with the same input features as the primary model. Maarastawi *et al.* (2018): 123 samples with environmental features distinct from those in the primary model. Bold means the best model per column.

## 4 Discussion

DL has rarely been applied to microbiome data due to two primary challenges: the results are difficult to interpret; and the properties of the data are often not favorable for DL. Interpretability of deep learning models is currently an open research topic of high interest (Manica *et al.*, 2019). Further, microbiome data are characterized by few samples in the same study (usually up to hundreds). Recent large-scale studies are beginning to address these limitations (Sayyari *et al.*, 2019), but they remain challenging. We address these as follows:

First, regarding DL interpretability, we provide a feature relevance analysis. In addition, given that we are solving 717 regression problems simultaneously, our performance metrics are aggregated. Following standard assessments for a unitary prediction problem would hinder interpretation of our results. To address this, we designed novel dis-aggregated assessments regarding the reliability of an individual microbe prediction (lowest error per OTU) and the reliability of an individual sample prediction (similarly colored patterns in microbial composition barplots).

Second, another challenge in microbiome data analyses is the low number of samples in an individual study. Our transfer learning proposal contributes a partial solution to that challenge, allowing the building of artificial intelligence (AI) predictive models even with small sample sizes. As we demonstrated, knowledge from the larger Walters *et al.* (2018) maize rhizosphere dataset, with thousands of samples, can be used to transfer predictive knowledge to the similar, but much smaller, Maarastawi *et al.* (2018) study. Moreover, transfer learning can also take advantage of distinct kinds of metadata, e.g. biotic and abiotic factors—in the case of this study, factors relevant to the soil microbiome, such as weather, chemical composition, etc.—combined in the microbiome deep latent space. In such a case, the performance of the prediction with the combined latent space may be expected to improve prediction performance compared to a latent space using just an isolated metadata source, such as the OTU latent space.

Our approach of creating reduced microbiome representations as a deep latent space could be extended to any other microbiome study type/site (e.g. grapevine, human gut, water, etc.). The requirement is to execute just one large-scale study per site-type, because DL approaches are only applicable (or recommendable) when thousands of samples are available. It is possible that rich knowledge in the study metadata for a particular case may reduce required scale of that initial study.

We note that the requirement to integrate thousands of samples from the same ecosystem/niche to build a widely-predictive model also points to a requirement for high-quality metadata that ensure the datasets are, in fact, comparable and interpretable. This requirement is not always met in practice. These concerns have also been raised by Sakowski *et al.* (2019), who note that there are few widely accepted quantitative predictive tools in microbiome research, in part due to a lack of standardization, reproducibility and accessibility of data and methods that would enable undertaking such studies. With the objective of ensuring our work is reproducible, and encouraging better transparency and data publication practices in the community, we have made all of our methods and result files available on GitHub via Jupyter notebooks.

Beyond technical reproducibility of a single study, we also detected more community-wide interoperability challenges. For example, between distinct microbiome datasets, due to a lack of standardization in OTU identification and, worse, in the taxonomic labeling/annotation, it is extremely difficult to map results from one study to another. In these cases, starting with the native data, transfer learning is entirely thwarted because there is no overlap in the microbe identifiers whose abundances we want to predict. In this study, we resolved this problem, after a difficult manual intersection between the 717 and 1900 OTUs in the two maize root microbiome datasets, successfully identifying very few common OTUs at different taxonomic levels; e.g. only 84 of 222 at Genus level (see additional data in Section 2.1). Such manual interventions are time-consuming, error prone, and ‘lossy’ (i.e. when a determination of identity cannot be made, the data must be discarded). There is a strong need for the microbiome community to act jointly to establish minimal standards for annotations and for study reporting.

## 5 Conclusions

This manuscript presents, and evaluates, a reproducible deep learning system capable of predicting the microbial composition of the maize rhizosphere starting from only environmental features. It outperforms other approaches in quantitative terms, in particular, providing the advantage of being capable of predicting the relative abundances of hundreds of bacterial species, rather than just a select few.

The computational contributions of our proposed microbiome AE include: (a) a novel dimensionality reduction approach to represent microbiome in a reduced latent space; (b) the ability to undertake challenging tasks in microbiome data analysis, such as to predict the microbial composition of hundreds of taxa based on a small number of features, rather than the more common (and straightforward) task of predicting a host phenotypic feature from the relative abundance of hundreds of taxa. In addition, this work makes the following contributions to the biological aspect of microbiome analysis: (c) the ability to predict microbiome composition based on a limited number of sequenced samples and/or a small number of relevant environmental variables; (d) the ability to predict more OTUs than previous approaches, thus enabling predictions at deeper taxonomic levels; and (e) the ability to predict hypothetical microbiomes that might arise in novel or predicted/designed ecosystems, e.g. under future climate change conditions or after an environmental insult such as a toxic spill.

Our AI system is also explainable. In this study, e.g. beyond the biological knowledge arising from an examination of the bacterial species composition, it revealed that the most relevant features to predict this microbial composition are plant age and precipitation. We are also able to identify which taxa are most accurately predicted, in order to be aware of any potential bias of the system.

We demonstrated that the knowledge in our encoded microbiome can be reused, via transfer learning, to gain insights in distinct but related studies, and by allowing complex analyses to be undertaken using fewer *de novo* sequencing samples. This will be useful in a wide range of cases where data are minimal or unavailable, or where resources or expertise limit sampling to only a few, easily accessible, environmental features.

This compressed representation opens-up many novel possibilities for microbiome data analysis, particularly with respect to knowledge retrieval and visualization. The condensed representation could be applied to any environment (gut, ocean, urban soil, etc.) where there is a representative set of samples available. The AE structure provides the ability to recover the complete abundance vector from codified samples in the deep latent space, making it possible to perform all analyses using the reduced coded data, and to recover the long vector only when required.

## Funding

Research was supported by the "Severo Ochoa Program for Centres of Excellence in R&D" from the Agencia Estatal de Investigación of Spain (grant SEV-2016-0672 (2017-2021)) to the CBGP. B.G.-J. was supported by a Postdoctoral contract associated to the Severo Ochoa Program. This work was supported by grants from Comunidad de Madrid (AGRISOST-CM S2018/BAA-4330 to J.M.) as well as UE Prima (PCI2019-103610 to J.M.).

*Conflict of Interest*: none declared.

## Supplementary Material

btaa971_Supplementary_Data
