# Supplementary material for: Predicting microbiomes through a deep latent space
Source: Bioinformatics. 2020 Dec 7;37(10):1444–51. doi: 10.1093/bioinformatics/btaa971 (PMC8208755; doi:10.1093/bioinformatics/btaa971)
Supplement: btaa971_Supplementary_Data [file btaa971_supplementary_data.pdf]

Supplementary Material for:
Predicting microbiomes through a deep latent space

Beatriz García-Jiménez , Jorge Muñoz , Sara Cabello , Joaquín Medina , and Mark D. Wilkinson

Contents

1 Methods **2**
1.1 Model architectures . . . 2
1.1.1 Reference prediction model . . . 2
1.1.2 Comparison models: Linear regression and Multi-Layer Perceptron (MLP) 2
1.2 Normalization . . . 3
1.3 Loss function . . . 3
1.4 Computational requirements . . . 4

2 Comparison with similar approaches **4**

3 Supplementary Figures **6**

1 Methods

Dataset: sample filtering

The initial number of OTUs from the study was 29,689, but the authors (Walters *et al.*, 2018) reduced this to 717 OTUs, corresponding to the OTUs shared in at least 80 percent of the samples, and samples with at least 10000 reads. We further filtered-out approximately 100 bulk soil samples.

1.1 Model architectures

1.1.1 Reference prediction model

Our prediction model takes environmental features as input and returns a prediction of the maize rhizosphere microbial composition. The model architecture is composed of two modules, as Figure S0C shows, sharing an intermediate knowledge representation of the microbiome, in a reduced code, called the microbiome latent space. Thus, the first module is a neural network (NN) taking environmental features as input and returning a microbiome latent representation. The second module is a decoder that takes that microbiome latent representation as input and returns a whole microbial composition.

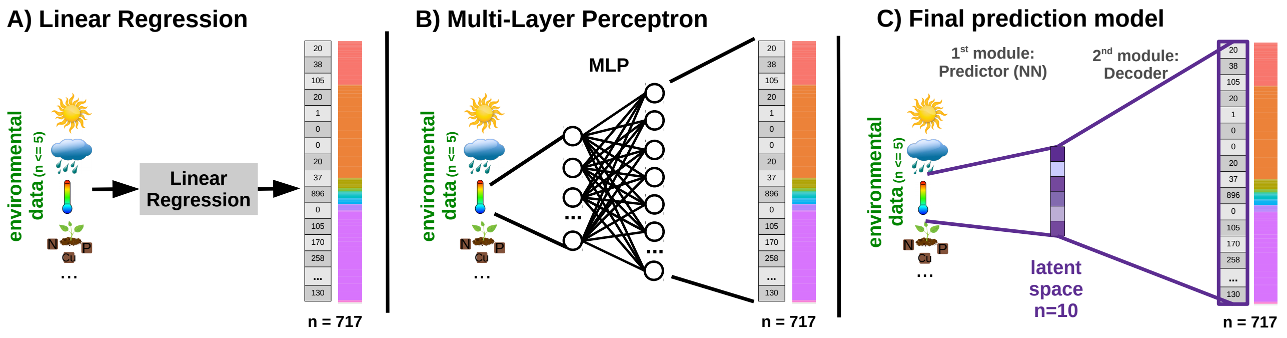

Figure S0: **Alternative model architectures.**

The input of our reference or selected model are three environmental variables: temperature, rain and plant age. The intermediate representation of our selected model is a OTU latent space of size 10, which comes from just OTUs. The output is the whole microbial composition of size 717, i.e. the relative abundance prediction of 717 OTUs. The first module has a structure of layers 3:128:64:32:16:10 (Input:IntermediateLayers:Output), while the second module, i.e. the decoder, has 10:256:512:717. The detailed technical definition of the architecture is available in the `reference_model_predictions_and_analysis.ipynb` Jupyter notebook on GitHub.

1.1.2 Comparison models: Linear regression and Multi-Layer Perceptron (MLP)

The input of the Linear Regression and Multi-Layer Perceptron models are subsets of the environmental variables (as described in the first column of Table 1), depending on the latent space model they are compared with. The output is the microbial abundances (Figure S0A and S0B).

The architecture and hyperparameters of the MLP were selected similarly to the predictor model (i.e. from environmental features to latent space prediction) of the AEs the MLP is compared with. The MLP has an architecture with 2 hidden layers of 128 and 512

(the best one found empirically). The input layer has variable size (depending on the subset of environmental features), and the output layer size is 717, i.e., the number of OTUs (no.env.variables:128:512:717 architecture). The encoder from the environmental features for the AE with combined latent space has an architecture no.env.variables:32:16:10 + decoder (256:512:717). The encoder for the AE with OTU latent space architecture was described in the previous section.

Additional details are provided in the Jupyter notebook documenting this study available on GitHub (`Notebooks/Auxiliary/model_reference_XdomainFeaturesXXX.ipynb` files, for example `model_reference_3domainFeatures.ipynb`).

1.2 Normalization

We selected two normalization approaches suitable to 16S microbiome absolute abundance data (Weiss *et al.*, 2017), and satisfying the constraints of our DL model based on an autoencoder, which requires inverse transformations to reconstruct the original input.

The first is Total-Sum normalization (TSS), i.e. relative abundances. This approach is commonly used in DL (Oh and Zhang, 2020), although it brings with several disadvantages, in that it does not remove compositionality (Aitchison, 1982). As such, several standard analyses cannot be applied without bias, such as comparative analysis between groups; however, these problems are distinct from how we use this approach in our study. For example, Gloor et al., 2017 recommends to avoid the use of Pearson correlations in compositional datasets, but this recommendation applies to computing the correlation between two features within the same dataset, while we compute correlations between features in different datasets: the real abundances and the predicted abundances. As such, this recommendation does not apply in this case.

The second approach is TSS followed by Centered Log Ratio (CLR). A logarithmic transformation is recommended to remove the compositionality of microbiome data (Zhou and Gallins, 2019). Because of the incompatibility of OTU table microbiome data sparsity (i.e. a high number of zeros) and logarithmic analyses, a small positive value is added to all abundances (in our case, pseudo-count = $1e^{-6}$). This normalization approach provides interesting advantages in our DL architecture. Although CLR can return negative values, the autoencoder accepts these as input, without substituting them with zeros (which, in other approaches, has the consequence of confounding them with the original structural zeros). Our AE architecture requires an inverse function, and this is provided by CLR in the form of Softmax (Pawlowsky-Glahn *et al.*, 2015). Moreover, Softmax is a standard activation function in DL architecture used to provide values similar to a probability distribution (all values add 1), simplifying the transformation of our output.

1.3 Loss function

We have used different loss functions in our experiments to guide the training.

- *Mean Squared Error (MSE).* The MSE is commonly used in AE as one the basic reconstruction error functions. It does not take into account information about the environmental features and it only tries to minimize the difference between the input and the output, giving more relevance to the bigger errors.

- *Crossentropy.* In our problem, it is more important to predict the proportions of each OTU rather the concrete values. So, if we use relative frequencies for the OTUs we can

use statistical distances as the Crossentropy. It has been used extensively for DL in the last years. For some experiments, we decided that the output of the AE was the relative frequency of the OTUs, which allowed us to use Crossentropy directly. For the AEs where we used the CLR for the normalization we apply the Softmax transformation to the output to get the relative frequencies.

- *Bray-Curtis dissimilarity.* Since we are analysing microbiome data, we also consider community ecology approaches as loss function, selecting a commonly used microbiome beta-diversity (i.e. between-samples distance) metric - the Bray-Curtis dissimilarity. Other metrics were considered to be the loss function but they are not differentiable.

1.4 Computational requirements

The experiments for hyper-parameter selection were run on a Linux server with two Nvidia 1080Ti GPUs with 11 GB of memory each, a Intel CPU 6900 with 16 cores and 32GB of RAM. Typical running times for each model selection experiment was approximately 5 minutes without full utilization of the GPU. We ran all experiments (more than 400 combinations) using different methods for multiprocessing in order to use both GPUs at 100% usage. It took less than 6 hours to run all hyper-parameter selection experiments. All other reported results were generated via the Jupyter Notebooks available in the project GitHub.

2 Comparison with similar approaches

Larsen *et al.*, 2012 explored how to predict microbial composition from environmental features; specifically they predicted an oceanic microbiome from environmental variables and interactions (between taxa and with the environment). One important difference when comparing that study with this one is that they reduced the number of taxa at the Species level (hundreds or thousands) by aggregating them at the Order level, resulting in 24 taxa. This resulted in a workable number of output variables, eliminating the need to address the dimensionality reduction problem. In our case, we are able to execute a similar analysis using hundreds of taxa, as shown in Table 2. In contrast to Larsen *et al.*, 2012, we use an Autoencoder architecture. Their intermediate representation was a matrix of interactions between the taxa themselves and between taxa and environmental features, retrieved with a Bayesian Network. The taxa prediction was implemented with a classical Artificial Neural Network, rather than novel DL techniques. Unfortunately, we cannot compare our results quantitatively because neither their data nor their software are available for reproducibility studies.

Ladau *et al.*, 2018 used regression models to establish the relationship between the abundances of the 53 most abundant families in a microbiome, and historical and contemporary climate variables. They concluded that climate change will increase diversity and change the relative abundances of soil bacteria.

From the methodological point of view, Xie *et al.* (2017) designed a similar DL architecture to ours. It is based on an Autoencoder, and is capable of making multiple regressions simultaneously. It is distinct, however, in that it was designed for an entirely different goal: to better understand the mechanisms involved in gene expression regulation. As such, we cannot compare our system - neither quantitatively nor qualitatively - with theirs.

The study most similar to ours is DeepMicro (Oh and Zhang, 2020). Here, we predict a microbiome code from environmental variables and then decode it to obtain the whole microbiome vector using an Autoencoder. DeepMicro also makes use of dimensionality reduction in

microbiome data via an Autoencoder architecture; however, the main difference between the approaches is their distinct goals. DeepMicro uses the code to predict human diseases, compared to our approach of predicting the code itself from environmental features, and decoding this code back to a microbiome. These distinct goals make it difficult to do a quantitative comparison of the two approaches. Additional differences include our representation of microbiome composition using only a species-level relative abundance profile, applying different numerical transformations (TSS or CLR), while DeepMicro also tested strain-level marker profiles (0/1 species absence/presence). Further, they use hundreds of samples, while we use thousands in our model training, as is recommended for DL where the objective is to achieve a good estimation of all the hyper-parameter values in the model architecture.

3 Supplementary Figures

Figure S1

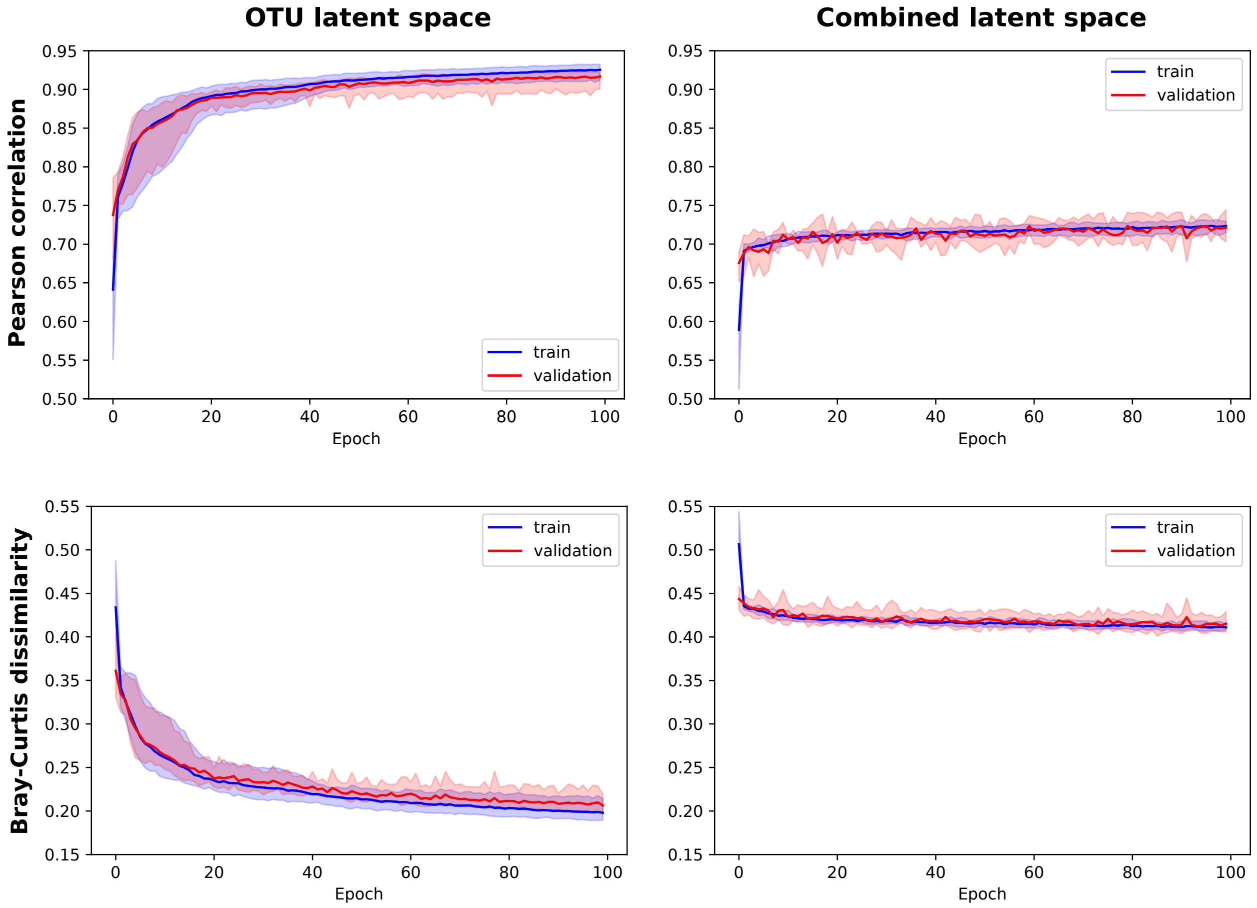

Figure S1: **Learning curves of autoencoder training.** The figure shows both autoencoders, with an OTU latent space and a combined latent space. Three environmental features of the reference model: Temperature, rain, plant age. The two performance metrics used in the current study are represented. In Pearson correlation, higher scores are better, because it is a correlation metric. In Bray-Curtis dissimilarity, lower scores are better, as it is a dissimilarity metric.

Multiple learning curves are represented in the pre-computed notebook results, in html files (for example, `https://github.com/jorgemf/DeepLatentMicrobiome/blob/master/Notebooks/OutputNotebooks/model_reference_3domainFeatures.html`)

Figure S2

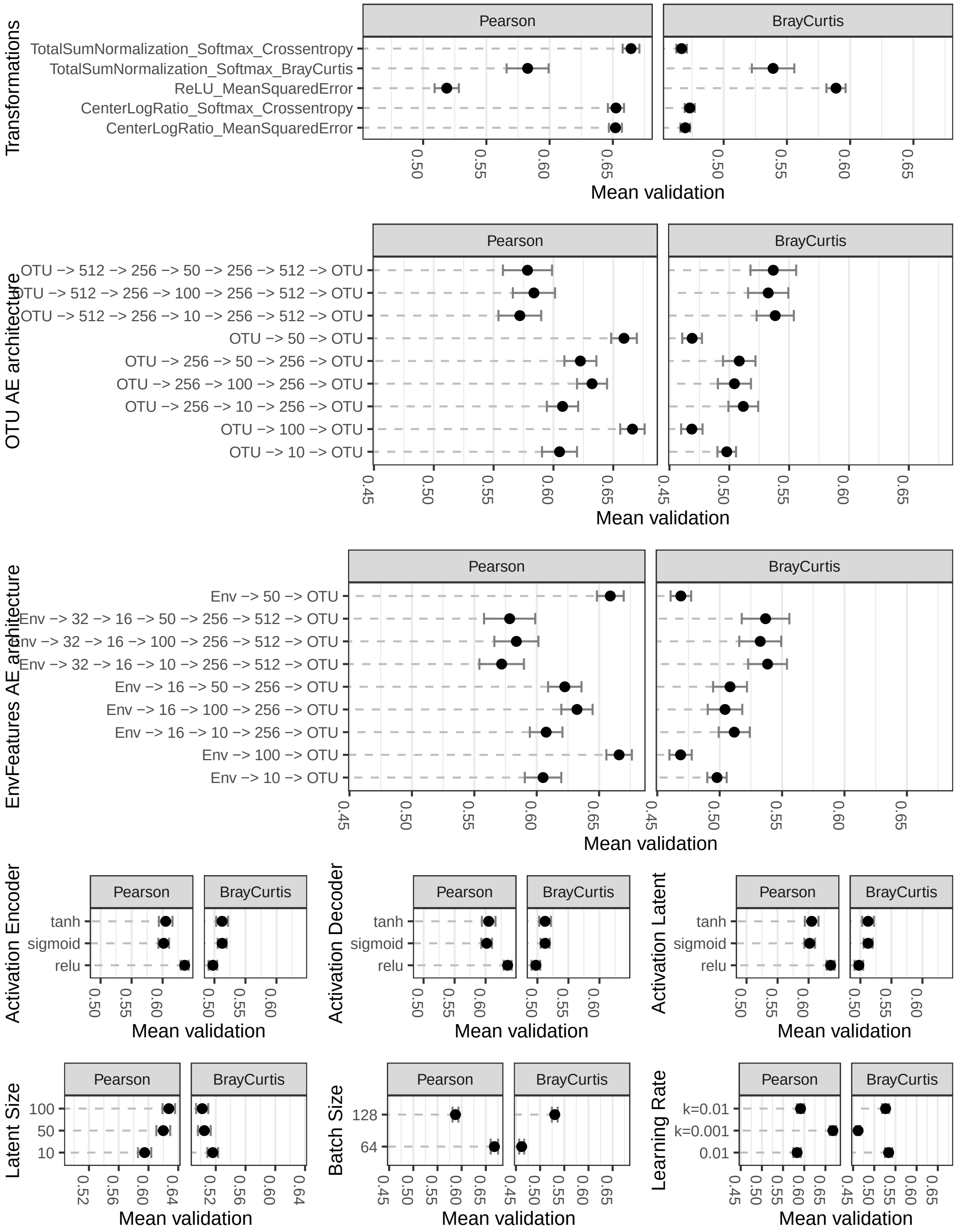

Figure S2: **Performance of different hyperparameter values.** Mean validation (5-fold CV on the training set) grouped by hyperparameter values. In Pearson correlation, higher scores are better, because it is a correlation metric. In Bray-Curtis dissimilarity, lower scores are better, as it is a dissimilarity metric. In 'Learning Rate' graph, k means constant.

Given that Pearson and Bray-Curtis are usually "mirrors" of one another, the best parameter value corresponds to those where the points in each graph-pair fall near the center of the graph-pair. In Transformation, Center Log Ratio and TSS works similarly. Regarding AE architectures, smaller autoencoders work better in general; however, we obtained the best results using larger autoencoders (more layers, more nodes per layer). There is little difference between the activation functions. The larger the latent space the better, although there is little difference between 50 and 100. Batch size of 64 with Learning rate of 0.001 works better than Batch size of 128 with Learning rate of 0.01.

Figure S3

Figure S3 shows that the performance of the reconstructed microbial composition (Y axes) is very high in both Pearson correlation, with most of the points close to the maximum correlation on the upper region, and Bray-Curtis dissimilarity, with most of the points close to the minimum dissimilarity in the lower region. The reconstructed performance is better than the performance of the microbial composition predicted from the environment, with points distributed over a wider area in the X axis), although most of the points are concentrated in the corners associated with the best performance in each measure (top-right and bottom-left, respectively).

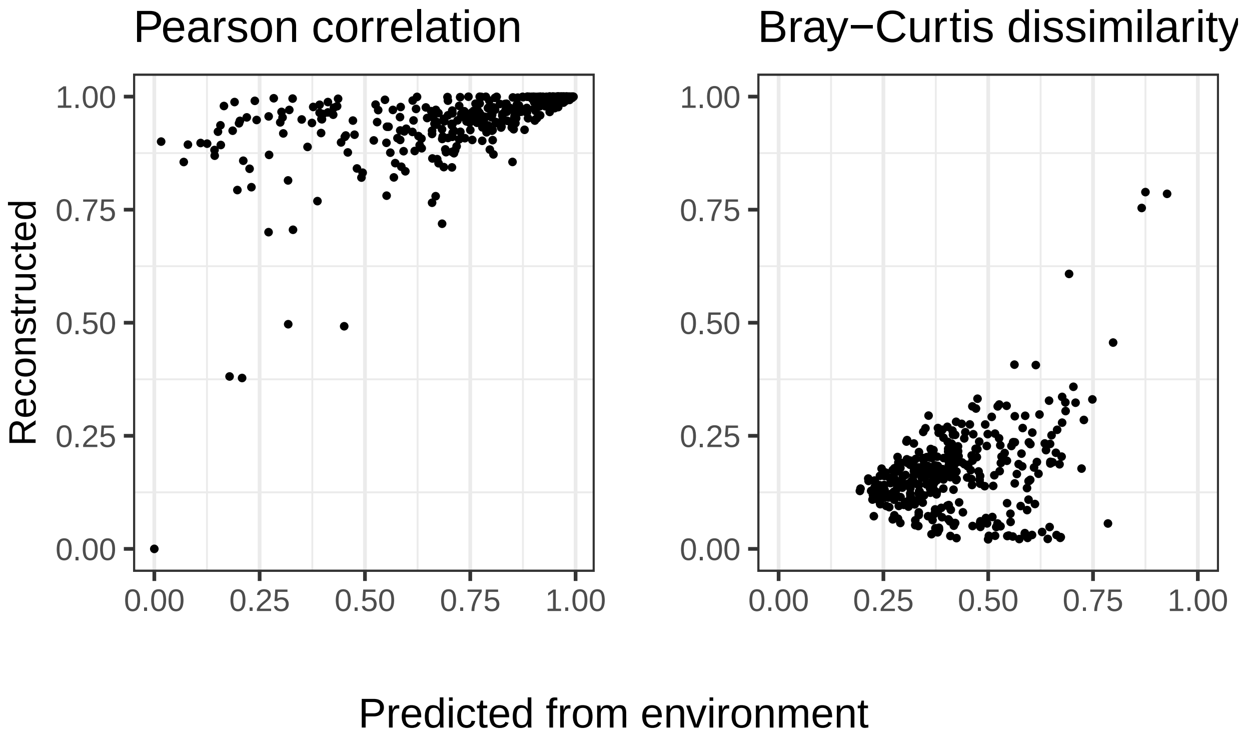

Figure S3: **Performance reference model per sample in test set.** Comparison of original abundances with those reconstructed from the autoencoder (Y axes) and original abundances with those predicted from the environmental features (X axes). In Pearson, higher scores are better, because it is a correlation metric. In Bray-Curtis, lower scores are better, as it is a dissimilarity metric. Both the autoencoder and the model from environmental features refer to the reference model (OTU latent space) selected and described in section 3.2.

Figure S4

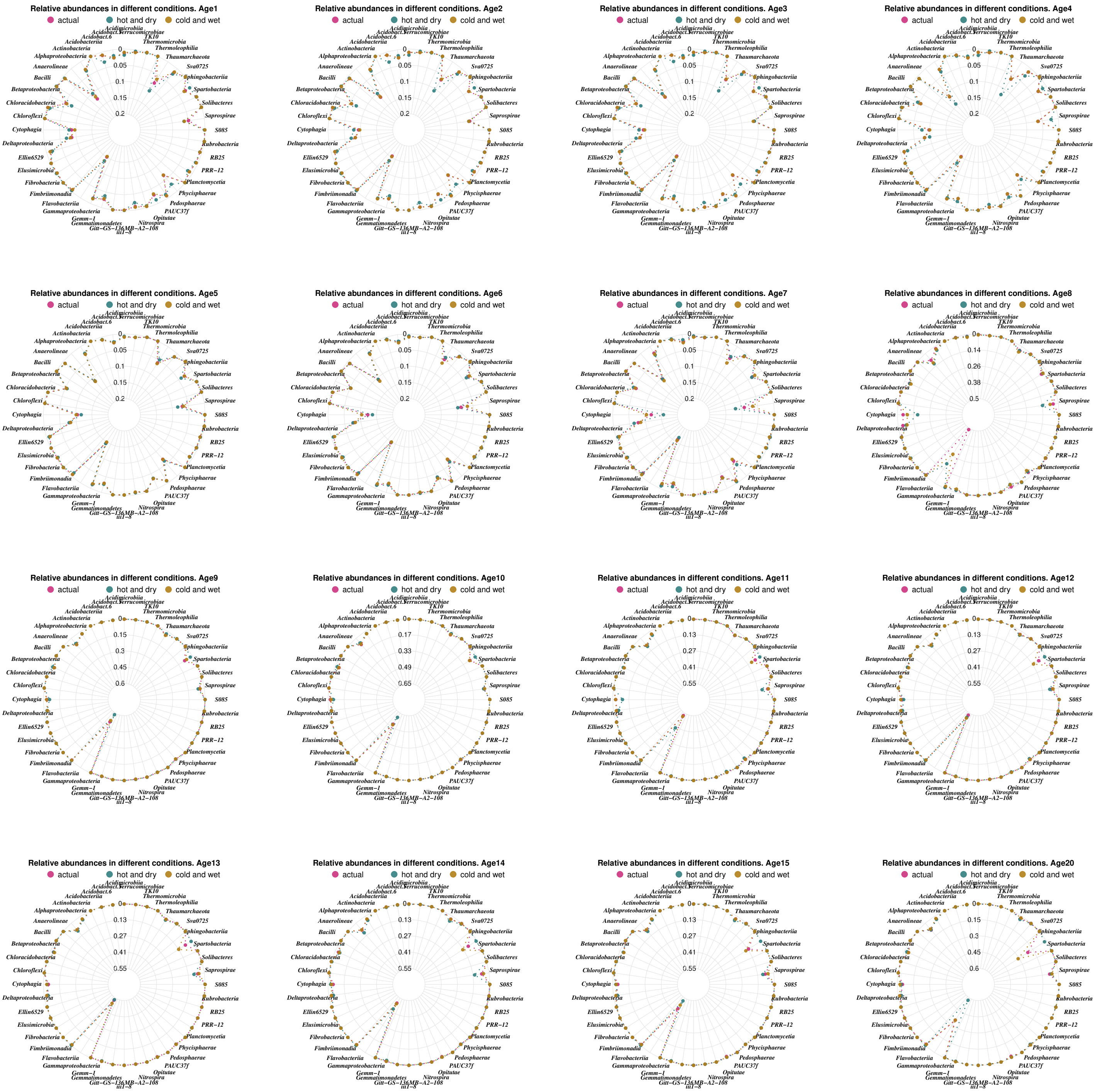

Figure S4: **Prediction of microbial composition in different predicted climate change conditions, at multiple plant ages.** Outcomes are reported at the Class taxonomic level. Each coloured point and dashed line indicates a sample in a different temperature/precipitation condition. ‘actual’: 59°F and 1.5 inches of rain; ‘hot and dry’: 86°F and 0 inches of rain; ‘cold and rain’: 50°F and 5 inches of rain. Note the difference in the maximum of relative abundance between different radar charts.

References

Aitchison, J. (1982). The Statistical Analysis of Compositional Data. *Journal of the Royal Statistical Society: Series B (Methodological)*, **44**(2), 139–160.

Ladau, J., Shi, Y., Jing, X., He, J.-S., Chen, L., Lin, X., Fierer, N., Gilbert, J. A., Pollard, K. S., and Chu, H. (2018). Existing Climate Change Will Lead to Pronounced Shifts in the Diversity of Soil Prokaryotes. *mSystems*, **3**(5).

Larsen, P. E., Field, D., and Gilbert, J. A. (2012). Predicting bacterial community assemblages using an artificial neural network approach. *Nature Methods*, **9**(6), 621–625.

Oh, M. and Zhang, L. (2020). DeepMicro: deep representation learning for disease prediction based on microbiome data. *Scientific Reports*, **10**(1), 6026.

Pawlowsky-Glahn, V., Egozcue, J. J., and Tolosana-Delgado, R. (2015). *Modeling and Analysis of Compositional Data*. wiley, Chichester, UK.

Walters, W. A., Jin, Z., Youngblut, N., Wallace, J. G., Sutter, J., Zhang, W., González-Peña, A., Peiffer, J., Koren, O., Shi, Q., Knight, R., Del Rio, T. G., Tringe, S. G., Buckler, E. S., Dangl, J. L., and Ley, R. E. (2018). Large-scale replicated field study of maize rhizosphere identifies heritable microbes. *Proceedings of the National Academy of Sciences of the United States of America*, **115**(28), 7368–7373.

Weiss, S., Xu, Z. Z., Peddada, S., Amir, A., Bittinger, K., Gonzalez, A., Lozupone, C., Zaneveld, J. R., Vázquez-Baeza, Y., Birmingham, A., Hyde, E. R., and Knight, R. (2017). Normalization and microbial differential abundance strategies depend upon data characteristics. *Microbiome*, **5**(1), 27.

Xie, R., Wen, J., Quitadamo, A., Cheng, J., and Shi, X. (2017). A deep auto-encoder model for gene expression prediction. *BMC Genomics*, **18**(Suppl 9), 845.

Zhou, Y.-H. and Gallins, P. (2019). A Review and Tutorial of Machine Learning Methods for Microbiome Host Trait Prediction. *Frontiers in Genetics*, **10**(JUN), 579.
